# ARC in Critical Ill OLT Recipients: True Prevalence, Risk Factors, and Impact on Early Aggressive PK/PD Target Non‐Attainment of CI Beta‐Lactam Therapy for Posttransplant Gram‐Negative Infections

**DOI:** 10.1111/tid.70237

**Published:** 2026-05-07

**Authors:** Milo Gatti, Riccardo De Paola, Matteo Rinaldi, Cristiana Laici, Antonio Siniscalchi, Pierluigi Viale, Federico Pea

**Affiliations:** ^1^ Department of Medical and Surgical Sciences Alma Mater Studiorum University of Bologna Bologna Italy; ^2^ Clinical Pharmacology Unit IRCCS Azienda Ospedaliero‐Universitaria di Bologna Bologna Italy; ^3^ Specialization School of Clinical Pharmacology and Toxicology Alma Mater Studiorum University of Bologna Bologna Italy; ^4^ Infectious Diseases Unit Department for Integrated Infectious Risk Management IRCCS Azienda Ospedaliero‐Universitaria di Bologna Bologna Italy; ^5^ Anesthesia and Intensive Care Medicine IRCCS Azienda Ospedaliero‐Universitaria di Bologna Bologna Italy

**Keywords:** augmented renal clearance, continuous infusion, beta‐lactams, early aggressive PK/PD target attainment, measured creatinine clearance, orthotopic liver transplant

## Abstract

**Background:**

To assess the true prevalence and the risk factors of augmented renal clearance (ARC) in critical orthotopic liver transplant (OLT) recipients and its impact on early aggressive pharmacokinetic/pharmacodynamic (PK/PD) target non‐attainment of continuous infusion (CI) beta‐lactams.

**Methods:**

OLT recipients without renal dysfunction and undergoing one or more measured creatinine clearance (mCLCr) assessments in the first 30 days posttransplant were retrospectively included. Reliability of three different eGFR formulas (Cockcroft–Gault, 2021, CKD‐EPI, and MDRD) in estimating mCLCr was tested. Univariate analyses compared the clinical features of ARC versus non‐ARC patients and the severity of the clinical conditions during ARC versus non‐ARC episodes in the whole cohort. In patients receiving CI beta‐lactam therapy, risk factors for early aggressive PK/PD target non‐attainment were investigated.

**Results:**

Among 450 critical OLT recipients, 112 were included. The true prevalence of ARC was 23.2%. ARC versus non‐ARC patients were younger (*p* = 0.003), had lower MELD score (*p* = 0.001), received lower number of intraoperative blood transfusions (*p* = 0.007), and had a lower need for venovenous bypass technique (*p* = 0.024). ARC episodes occurred more frequently in patients having lower SOFA score (*p* = 0.005) and lower median serum creatinine levels (*p* < 0.001). None of the eGFR formulas properly estimated mCLCr. In the subset of 50 OLT recipients receiving 60 CI beta‐lactam treatment courses, ARC was the only factor associated with early aggressive PK/PD target non‐attainment (*p* = 0.018).

**Conclusions:**

ARC is a quite prevalent condition among critical OLT recipients, which may hinder early aggressive PK/PD target attainment when using standard beta‐lactams dosing regimens, regardless of delivery by CI.

## Graphical abstract

Augmented renal clearance (ARC) represents a quite prevalent condition among critical orthotopic liver transplant recipients (23.2%). ARC is associated with different demographics and clinical features and significantly affects the attainment of aggressive beta‐lactams pharmacokinetic/pharmacodynamic targets despite the administration of standard dosing regimens by continuous infusion.

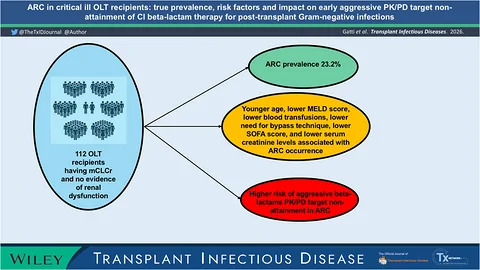

**Abbreviations**
ARC: augmented renal clearance
AUC: area under curve
BL: beta‐lactam
BL/BLIcbeta‐lactam/beta‐lactamase inhibitor combination: beta‐lactam/beta‐lactamase inhibitor combination
BLI: beta‐lactamase inhibitor
BMI: body mass index
CG: Cockcroft–Gault
CI: continuous infusion
CLCr: creatinine clearance
CRRT: continuous renal replacement therapy
DCD: donation after circulatory death
eGFR: estimated glomerular filtration rate
ICU: intensive care unit
IQR: interquartile range
mCLCr: measured creatinine clearance
MDRD: modification of diet in renal disease
MELD: Model for End‐Stage Liver Disease
MIC: minimum inhibitory concentration
OLT: orthotopic liver transplant
OR: odds ratio
PK/PD: pharmacokinetic/pharmacodynamic
SOFA: sequential organ failure assessment
TDM: therapeutic drug monitoring

## 1 Introduction

Orthotopic liver transplant (OLT) is currently the established standard of care for end‐stage liver disease. In recent years, both clinical cure and survival rates of OLT recipients have been remarkably increased, largely because of improvements in the capabilities of both recognizing earlier the posttransplant complications and managing them properly with effective treatments [1]. Augmented renal clearance (ARC) is a pathophysiological phenomenon occurring mainly in critically ill patients, and is characterized by an increase in creatinine clearance (CLCr) over physiological values, namely ≥ 120–130 mL/min [2]. Recent studies showed that the proportion of ARC could be rather high in several different settings of critically ill patients having no evidence of renal dysfunction. Noteworthy, it ranged from 39.5% to 56% in hyperdynamic septic patients to 65% in major burn patients, 85.7% in polytrauma patients, up to 100% in neurocritical patients [2, 3, 4, 5]. Importantly, knowing ARC prevalence in a given setting of critically ill patients may be therapeutically relevant, since ARC, by enhancing the elimination of renally cleared drugs, could potentially place critically ill patients at high risk of therapeutic failure when using standard doses of some hydrophilic drugs [6, 7]. Measuring creatinine clearance (mCLCr) rather than using predictive formulas of estimated glomerular filtration rate (eGFR) may be fundamental in preventing misestimation of the ARC prevalence among critically ill patients without any evidence of renal dysfunction [8, 9].

To the best of our knowledge, the true prevalence of ARC established by means of the mCLCr is still unknown among OLT recipients in the early‐posttransplant period. Acquiring such information would be therapeutically relevant considering the quite frequent need of some hydrophilic drugs, namely the beta‐lactam (BL), for treating early posttransplant Gram‐negative bacterial infections in this setting [10].

BLs are time‐dependent agents whose efficacy is based traditionally on concentrations persisting for 100% of time above the minimum inhibitory concentration (MIC) of the pathogen (100% T_>MIC_) [11]. Indeed, recent studies highlighted that this conservative pharmacokinetic/pharmacodynamic (PK/PD) target could not suffice for optimal treatment of critically ill patients [12, 13]. A recent meta‐analysis showed that when treating Gram‐negative infections in the critically ill patients attaining an aggressive PK/PD target, defined as 100% T_>4xMIC_, was significantly associated with both higher clinical cure rate (odds ratio [OR] 1.69; 95% CI: 1.15–2.49) and lower risk of BL resistance development (OR 0.06; 95% CI: 0.01–0.29) compared to the conservative one [13]. Importantly, ARC was found to be a significant independent predictor of aggressive PK/PD target non‐attainment, whereas conversely prolonged/continuous infusion (CI) administration of BLs resulted as a protective factor [13].

This retrospective study was aimed to assess the true prevalence of ARC episodes in a large cohort of critical OLT recipients having mCLCr in the first 30‐day posttransplant, the potential risk factors associated with ARC, and the reliability of some formulas to calculate eGFR in predicting ARC. In addition, the impact of ARC on early aggressive PK/PD target non‐attainment of BLs was assessed in a subset of OLT recipients receiving CI BL treatment because of early posttransplant Gram‐negative bacterial infections.

## 2 Methods

OLT recipients admitted to the posttransplant intensive care unit (ICU) of the IRCCS Azienda Ospedaliero‐Universitaria of Bologna between June 2021 and May 2025, having mCLCr in the first 30‐day posttransplant period, were retrospectively included. Patients having evidence of renal dysfunction, namely with a baseline serum creatinine level ≥ 1.5 mg/dL and/or undergoing continuous renal replacement therapy (CRRT), were excluded because of the risk of misestimating eGFR values based on CLCr predictive formulas [8].

Demographic (age, sex, weight, height, and body mass index [BMI]) and clinical/laboratory data (underlying liver disease requiring transplantation, pre‐OLT Model for End‐Stage Liver Disease [MELD] score, donor type including donation after circulatory death [DCD], and performance of combined liver–kidney transplantation) were collected for each patient. Intraoperative variables (i.e., surgery duration, intraoperative fluid balance, number of blood product transfusions, execution of biliodigestive anastomosis, bypass requirement, intraoperative serum lactate, need for vasopressors, post‐reperfusion syndrome occurrence, and requirement for open abdomen presence at the end of surgical intervention) were also retrieved.

The mCLCr was calculated by comparing creatinine levels in a 24‐h urine collection to serum levels based on the formula detailed in Table S1. Variables useful at assessing the severity of the clinical conditions were retrieved at each occasion of mCLCr assessment (i.e., sequential organ failure assessment [SOFA] score, need of mechanical ventilation and/or of vasopressor support, serum creatinine). ARC was defined as a mCLCr value ≥ 130 mL/min in males and ≥ 120 mL/min in females [2]. The reliability of three different eGFR formulas as a surrogate of mCLCr, namely the Cockcroft–Gault (CG) [14, 15], the 2021 CKD‐EPI [16], and the modification of diet in renal disease (MDRD) [17] (reported in Table S1), was also tested.

In a subset of OLT recipients receiving a therapeutic drug monitoring (TDM)‐guided therapy with a CI BL (namely piperacillin‐tazobactam, ceftazidime, meropenem, ceftolozane‐tazobactam, ceftazidime‐avibactam, meropenem‐vaborbactam, or cefiderocol) as empirical or targeted treatment of an early posttransplant Gram‐negative bacterial infection, also the impact of ARC on early aggressive PK/PD target non‐attainment was assessed at the first TDM assessment during treatment with standard doses. In these cases, BL therapy was always started with a loading dose (namely, 9, 2, 2, 2/1, 2/0.5, 2/2, or 2 g over 2‐h infusion for piperacillin‐tazobactam, ceftazidime, meropenem, ceftolozane‐tazobactam, ceftazidime‐avibactam, meropenem‐vaborbactam, or cefiderocol, respectively) followed by an initial standard CI maintenance dose (namely, 16/2 g q24h, 6 g q24h, 0.5–1 g q6h, 9 g q24h, 2 g/0.5 g q8h, 2 g/2 g q8h, and 2 g q8h for piperacillin‐tazobactam, ceftazidime, meropenem, ceftolozane‐tazobactam, ceftazidime‐avibactam, meropenem‐vaborbactam, or cefiderocol, respectively). CI administration was granted on the basis of the different stability restrictions of each BL agent, and aqueous solutions were reconstituted accordingly (i.e., every 6–8 h for meropenem and/or meropenem‐vaborbactam, every 8 h for ceftazidime‐avibactam and cefiderocol, and every 24 h for piperacillin‐tazobactam, ceftazidime, and ceftolozane‐tazobactam) [18]. Total plasma steady‐state concentrations (C_ss_) of each BL were measured by means of different validated chromatography–tandem mass spectrometry methods [19, 20, 21, 22, 23]. The free (*f*) C_ss_ were calculated from the total ones based on the specific plasma protein binding rate reported in the literature for each agent (namely 20% for piperacillin, 10% for ceftazidime, 2% for meropenem, 20% for ceftolozane, 59% for cefiderocol, 23% for tazobactam, 7% for avibactam, and 33% for vaborbactam) [24, 25, 26, 27, 28, 29]. Early aggressive PK/PD target attainment of BL was assessed at the first TDM assessment, namely within 72 h from starting therapy, while the patients were receiving standard CI maintenance dosing regimens. For the BLs (namely ceftazidime, meropenem, and cefiderocol), the aggressive PK/PD target attainment was defined as a *f*C_ss_/MIC ratio > 4 [30]. For the beta‐lactam/beta‐lactamase inhibitor combinations (BL/BLIc) (namely piperacillin‐tazobactam, ceftolozane‐tazobactam, ceftazidime‐avibactam, and meropenem‐vaborbactam), the aggressive PK/PD target attainment was joint and defined as a BL *f*C_ss_/MIC ratio > 4 coupled with a BLI *f*C_ss_/target concentration (C_T_) ratio > 1 for tazobactam and avibactam, or a free area under the concentration‐time curve (*f*AUC)/MIC ratio > 24 for vaborbactam [30]. The C_T_ corresponded to the fixed BLI concentration adopted by the EUCAST for testing the in vitro susceptibility of each BL/BLIc, namely 4 mg/L for tazobactam and avibactam or 8 mg/L for vaborbactam.

The MIC value inserted for calculating the BL *f*C_ss_/MIC ratio was the precise MIC value of the clinical isolate in the case of targeted therapy or the EUCAST clinical breakpoint against the suspected Gram‐negative pathogen in case of empirical treatment (namely 2 mg/L for meropenem, 4 mg/L for ceftolozane‐tazobactam, and 8 mg/L for piperacillin‐tazobactam, ceftazidime, ceftazidime‐avibactam, or meropenem‐vaborbactam).

The demographics and clinical features of the included OLT recipients were summarized by using the median and interquartile range (IQR) for continuous variables, whereas absolute frequencies and percentages were adopted for categorical variables.

A dual approach was adopted for addressing those patients having multiple mCLCr measurements over time. Specifically, in the analysis concerning patients’ features, each patient was counted individually and was considered as having ARC if experienced at least one episode. Conversely, in the analysis concerning ARC episodes, all the mCLCr measurements performed in each patient were included.

Univariate analyses (by means of the Fisher's exact test or the chi‐squared test for categorical variables and of the Mann–Whitney *U* test for continuous variables) were performed for comparing the clinical features of ARC versus non‐ARC patients and the severity of the clinical conditions during ARC versus non‐ARC episodes in the whole cohort of OLT recipients. In addition, it was used for comparing the impact of ARC and/or of the type of BL treatment on early aggressive PK/PD target attainment or non‐attainment in the subset of patients receiving BL therapy.

The relationships between the mCLCr and the eGFR_CG_, eGFR_CKD‐EPI_, and the eGFR_MDRD_ were assessed by means of linear regression analysis. All analyses were performed using MedCalc for Windows (MedCalc statistical software, version 19.6.1, MedCalc Software Ltd., Ostend, Belgium). Statistical significance was defined as a two‐tailed *p* value < 0.05.

## 3 Results

A total of 450 patients undergoing OLT during the study period were screened. Among the 151 of them having one or more mCLCr assessments during the first 30 posttransplant days, 112 had serum creatinine levels < 1.5 mg/dL and did not undergo CRRT, so they were finally included in the study. Overall, the true ARC prevalence was 23.2% (26/112) (Figure S1), and the first ARC episode occurred within 7 days from transplantation in the vast majority of cases (20/26; 76.9%). ARC prevalence in the first, second, third, and fourth week after OLT was 17.9% (20/112), 8.9% (10/112), 3.6% (4/112), and 2.7% (3/112), respectively.

The demographic and clinical characteristics of the overall study population and of the ARC versus non‐ARC patients compared by univariate analysis are reported in Table 1. In the overall population, the median (IQR) age was 58.5 years (52.5–65.0) with a slight male prevalence (65.2%). HCV‐related cirrhosis was the most prevalent underlying liver disease (16 cases; 14.3%), followed by alcoholic cirrhosis (15 cases; 13.4%), primary sclerosing cholangitis (11 cases; 9.8%), and polycystic disease (10 cases; 8.9%). The median (IQR) MELD score was 13.6 (9.8–21.9). DCD was applied in 20 cases (17.9%), combined kidney–liver transplantation in 8 (7.1%), and re‐transplantation in the other 8 (7.1%). A venovenous bypass technique was needed during OLT in 21 cases (18.8%). At the univariate analysis, ARC compared to non‐ARC patients were younger (median age 54.5 vs. 61.0 years; *p* = 0.003), had a lower MELD score at transplantation (median 10.3 vs. 15.4; *p* = 0.001), received a lower number of intraoperative blood transfusions (median 2 vs. 4 units; *p* = 0.007), and had a lower need of venovenous bypass technique during OLT (3.8% vs. 23.3%; *p* = 0.024).

**TABLE 1:** Demographics and clinical characteristics comparing OLT recipients exhibits or not ARC.

Demographics and clinical variables	Overall (*n* = 112)	ARC (*n* = 26)	No ARC (*n* = 86)	Univariate analysis *p* value
Patient demographics				
Age (years) (median [IQR])	58.5 (52.5–65.0)	54.5 (48.25–61.0)	61.0 (53.25–67.0)	**0.003**
Gender (male/female) (*n* [%])	73/39 (65.2/34.8)	17/9 (65.4/34.6)	56/30 (65.1/34.9)	0.98
Body weight (kg) (median [IQR])	71.0 (59.0–80.0)	73.0 (58.0–80.0)	70.0 (59.25–80.0)	0.74
Body mass index (kg/m^2^) (median [IQR])	24.4 (21.6–27.6)	24.5 (21.5–27.1)	24.4 (21.6–27.6)	0.80
OLT features				
MELD score at transplantation (median [IQR])	13.6 (9.8–21.9)	10.3 (6.8–12.7)	15.4 (10.8–23.8)	**0.001**
Donation after circulatory death (*n* [%])	20 (17.9)	7 (26.9)	13 (15.1)	0.17
Combined kidney–liver transplant (*n* [%])	8 (7.1)	0 (0.0)	8 (9.3)	0.19
Re‐OLT (*n* [%])	8 (7.1)	2 (7.7)	6 (7.0)	0.99
Underlying liver disease (*n* [%])				
HCV	16 (14.3)	6 (23.0)	10 (11.6)	0.19
Alcoholic cirrhosis	15 (13.4)	2 (7.7)	13 (15.1)	0.51
Primary sclerosing cholangitis	11 (9.8)	3 (11.5)	8 (9.3)	0.71
Polycystic disease	10 (8.9)	0 (0.0)	10 (11.6)	0.11
Liver cancer	9 (8)	3 (11.5)	6 (7)	0.43
Autoimmune cirrhosis	7 (6.2)	3 (11.5)	4 (4.7)	0.35
Alcoholic + dysmetabolic cirrhosis + liver cancer	6 (5.3)	1 (3.8)	5 (5.8)	0.99
HBV	5 (4.5)	2 (7.7)	3 (3.5)	0.32
HDV + HBV	4 (3.6)	2 (7.7)	2 (2.3)	0.23
HBV + alcoholic + dysmetabolic cirrhosis	3 (2.7)	0 (0.0)	3 (3.5)	0.99
Dysmetabolic cirrhosis	3 (2.7)	0 (0.0)	3 (3.5)	0.99
HBV + HCV	3 (2.7)	1 (3.8)	2 (2.3)	0.55
Cryptogenic cirrhosis	3 (2.7)	0 (0.0)	3 (3.5)	0.99
Subacute hepatitis	3 (2.7)	1 (3.8)	2 (2.3)	0.55
Chronic rejection	1 (0.9)	0 (0.0)	1 (1.2)	0.99
Others	13 (11.6)	2 (7.7)	11 (12.8)	0.73
Surgical features				
OLT duration (minutes) (median [IQR])	445.0 (390.0–537.5)	465.0 (395.0–553.8)	445.0 (390.0–510.0)	0.76
Biliodigestive anastomosis (*n* [%])	25 (22.3)	7 (26.9)	18 (20.9)	0.52
Intraoperative blood transfusions (units) (median [IQR])	3 (1–6)	2 (0–2.75)	4 (2–7)	**0.007**
Intraoperative fluid balance (mL) (median [IQR])	6800 (5600–8150)	7050 (5450–8050)	6800 (5700–8100)	0.87
Vasopressor support (mcg/Kg/min) (median [IQR])	0.10 (0.0–0.30)	0.02 (0–0.15)	0.12 (0.0–0.33)	0.08
Need of venovenous bypass during OLT (*n* [%])	21 (18.8)	1 (3.8)	20 (23.3)	**0.024**
Intraoperative serum lactate (mmol/L) (median [IQR])	1.5 (1.1–2.8)	1.45 (1.125–1.775)	1.6 (1.0–3.0)	0.24
Post‐reperfusion syndrome (*n* [%])	57 (50.9)	16 (61.5)	41 (47.7)	0.22
Open abdomen (*n* [%])	1 (0.9)	0 (0.0)	1 (1.2)	0.99

*Note*: Bold values are statistically significant.

Abbreviations: ARC, augmented renal clearance; IQR, interquartile range; MELD, model for end‐stage liver disease; OLT, orthotopic liver transplant.

Among the 26 ARC patients, 11 (42.3%) experienced multiple ARC episodes during the first 30 posttransplant days (namely, 6 had 2 episodes, 2 had 4 episodes, and 1 each had 3, 7, and 9 episodes) for a total of 54 episodes (timeline of all ARC episodes in Figure S2). The overall median (IQR) mCLCr was of 153.7 mL/min (139.1–210.8 mL/min). Univariate analysis comparing ARC‐ versus non‐ARC episodes showed that ARC episodes (Table S2) were associated with lower SOFA score (median 5 vs. 6 points; *p* = 0.005) and lower serum creatinine levels (median 0.45 vs. 0.77 mg/dL; *p* < 0.001).

Linear regression analyses of the relationships between the mCLCr and the eGFR calculated by means of the three different equations showed only weak to moderate correlations (Figure 1), with eGFR_CG_ (*R* ^2^ = 0.57; panel A) and eGFR_MDRD_ (*R* ^2^ = 0.43; panel B) overestimating, and eGFR_CKD‐EPI_ (*R* ^2^ = 0.36; panel C) underestimating the mCLCr. Consequently, compared to the true prevalence (23.2%), the presumed prevalence of ARC was overestimated by both the eGFR_CG_ and the eGFR_MDRD_ (31.3% and 32.1%, respectively), and underestimated by the eGFR_CKD‐EPI_ (8.9%). Figure 2 depicts the temporal trend of the mean daily mCLCr during the ARC episodes compared with the mean eGFR_CG_, eGFR_MDRD_, and eGFR_CKD‐EPI_.

**FIGURE 1:**
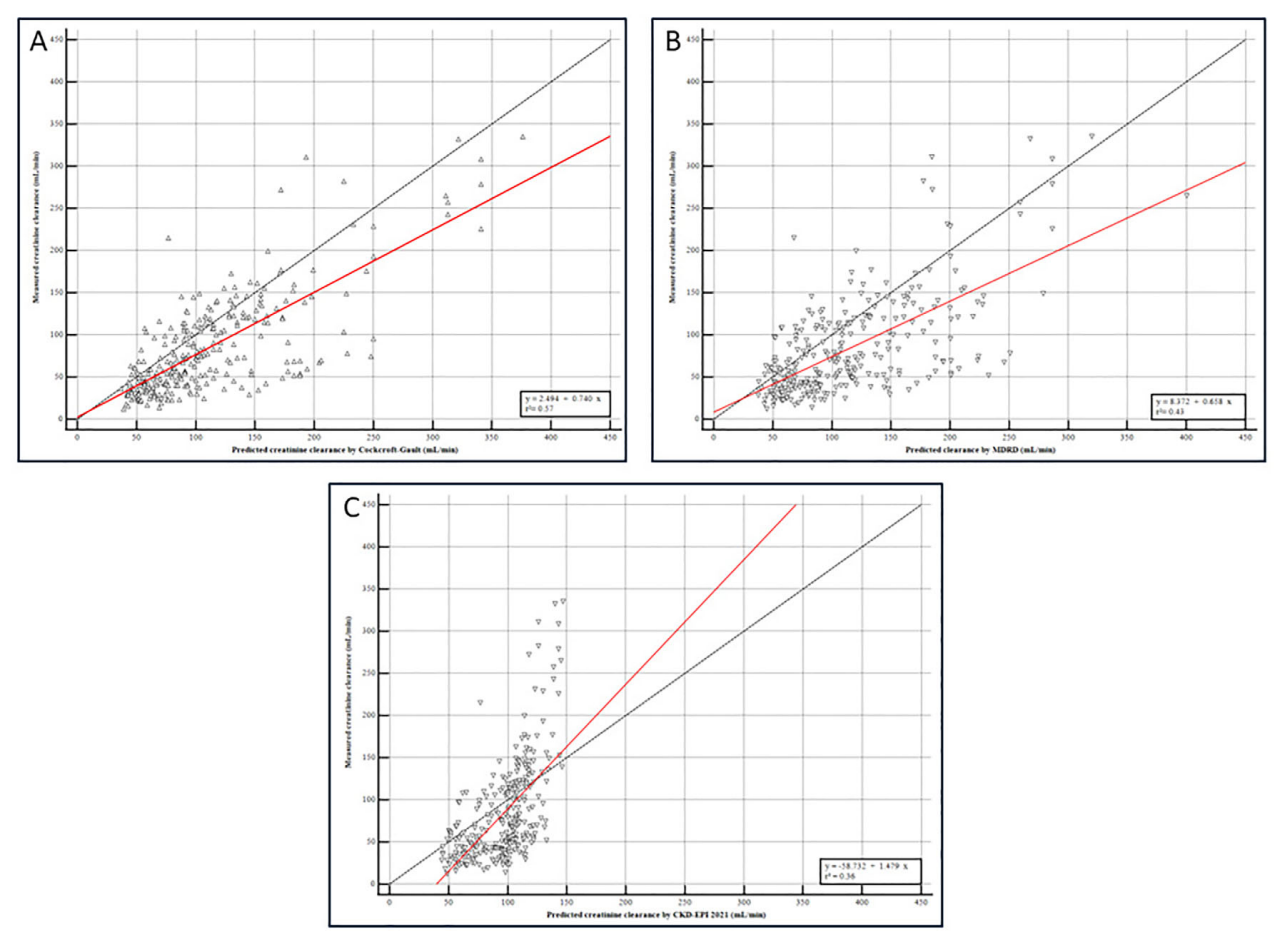
Linear regression between measured creatinine clearance (mCRC_L_) and estimated creatinine clearance according to the Cockcroft–Gault (A), MDRD (B), and CKD‐EPI 2021 (C) equations in ARC and non‐ARC patients. Scatterplots display individual measurements. Regression lines and *R* ^2^ values are reported for each equation.

**FIGURE 2:**
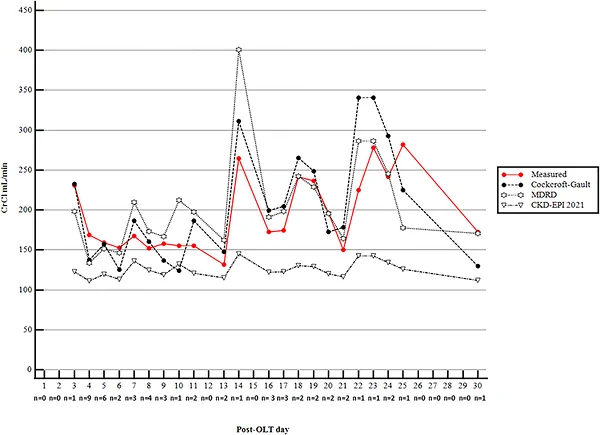
Temporal trend of both mean daily mCLCr and eGFR calculated by means of the Cockcroft–Gault (A), MDRD (B), and CKD‐EPI 2021 (C) equations in ARC OLT recipients in the first 30‐day posttransplant period.

A subset of 50 OLT recipients (44.6%) received one or more TDM‐guided courses of CI BL treatment because of early‐posttransplant Gram‐negative bacterial infections occurring in the first 30‐posttransplant days, for a total of 60 (Figure S3). Among these latter, 32 were empirical (5 during ARC and 27 during non‐ARC episodes, respectively) and 28 were targeted (10 during ARC and 18 during non‐ARC episodes, respectively). Meropenem was the most frequently used BL (26 treatment courses), followed by piperacillin‐tazobactam (24 treatment courses), meropenem‐vaborbactam (4 treatment courses), ceftazidime‐avibactam (3 treatment courses), ceftazidime, ceftolozane‐tazobactam, and cefiderocol (1 treatment course each). The proportion of early aggressive PK/PD target non‐attainment of BLs at the first TDM assessment while undergoing standard dosing regimens was 13.3% (8/60, namely 4 with piperacillin/tazobactam, 3 with meropenem, and 1 with meropenem/vaborbactam). Univariate analysis comparing attainment versus non‐attainment showed that ARC was the only factor associated with early aggressive PK/PD target non‐attainment (19.2% vs. 62.5%; *p* = 0.018) (Table 2).

**TABLE 2:** Univariate analysis comparing OLT recipients having early aggressive PK/PD target attainment versus non‐attainment.

Variables	Early aggressive PK/PD target attainment (*n* = 52)	Early aggressive PK/PD target non‐attainment (*n* = 8)	Univariate analysis *p* value
ARC	10 (19.2)	5 (62.5)	**0.018**
Empirical	30 (57.7)	2 (25.0)	0.13
Targeted	22 (42.3)	6 (75.0)	0.13
Beta‐lactams	25 (48.1)	3 (37.5)	0.71
Meropenem MD 0.5 g q6h	18 (34.6)	2 (25.0)	0.71
Meropenem MD 1 g q6h	5 (9.6)	1 (12.5)	0.99
Ceftazidime	1 (1.9)	0 (0.0)	0.99
Cefiderocol	1 (1.9)	0 (0.0)	0.99
Beta‐lactam/beta‐lactamase inhibitor combinations	27 (51.9)	5 (62.5)	0.71
Piperacillin‐tazobactam	20 (38.5)	4 (50.0)	0.70
Ceftolozane‐tazobactam	1 (1.9)	0 (0.0)	0.99
Ceftazidime‐avibactam	3 (5.8)	0 (0.0)	0.99
Meropenem‐vaborbactam	3 (5.8)	1 (12.5)	0.44

*Note*: Bold value is statistically significant.

Abbreviations: ARC, augmented renal clearance; MD, maintenance dosing; PK/PD, pharmacokinetic/pharmacodynamic.

## 4 Discussion

To the best of our knowledge, this is the first study that investigated the true prevalence of ARC in a large cohort of critically ill OLT recipients and that assessed its impact on early aggressive PK/PD target non‐attainment of CI BLs in the subset of those receiving therapy because of early posttransplant Gram‐negative infections. Overall, the findings showed that around one‐fifth of OLT recipients without renal dysfunction experienced one or more ARC episodes in the first 30‐day posttransplant, especially during the first week. In addition, they showed that this condition may cause early aggressive PK/PD target non‐attainment of BLs when using standard dosing regimens for treating early posttransplant Gram‐negative infections, regardless of CI administration.

The true prevalence of ARC in this setting of critically ill patients was lower than that observed in other studies in which ARC was really documented by means of mCLCr assessments. To this regard, a prospective observational study conducted among 80 neurocritical patients affected by intracerebral hemorrhage or aneurysmal subarachnoid hemorrhage found a prevalence of true ARC of 50.0% and 94.0%, respectively, with a mean mCLCr of 119.5 ± 57.2 mL/min and 147.9 ± 50.2 mL/min [8]. Similarly, a prospective observational study conducted among 205 neurocritical patients reported a prevalence of true ARC of 82.0% [3]. A prevalence of true ARC of 78.0% was found among 74 critical patients affected by traumatic brain injury or subarachnoid hemorrhage, with a median (IQR) mCLCr of 176 mL/min (135–214 mL/min) [31]. Finally, a retrospective analysis conducted among 200 critical polytrauma patients reported a prevalence of true ARC of 61.0%, with a median (IQR) mCLCr of 184 mL/min (141–233 mL/min) [9]. Beyond this, likewise to what was observed in the aforementioned other settings of critically ill patients [3, 8, 9, 31], the severity of the underlying clinical conditions immediately after the triggering event, namely the OLT transplant in our study, could have had a major role in affecting the appearance of ARC. The vast majority of the ARC OLT recipients experienced the first episode in the very early posttransplant period, and one had two episodes during the first posttransplant week. ARC patients were younger, had lower MELD scores at transplantation, and needed a lower number of intraoperative blood transfusions and a lower use of the venovenous bypass technique during the intervention. It could be speculated that in the younger critically ill OLT recipients having less severe comorbid conditions, ARC occurrence could be favored by a surplus of renal reserve, as just previously postulated for other conditions in other special populations of critically ill patients [32]. Regarding younger age as being a risk factor of ARC, this is in agreement with other studies carried out in other types of critically ill patients, namely polytrauma, septic, and/or trauma brain injury patients [4, 5, 7]. Regarding the severity of the clinical conditions, it may be speculated that having better clinical conditions in the peri‐ and/or early posttransplant period could prevent renal failure occurrence and could theoretically favor the appearance of ARC in the immediate post‐OLT period. This could be hypothesized based on some previous indirect findings. In a retrospective study, the use of venovenous bypass technique during transplantation was found to be an independent predictor of risk of renal failure in the early post‐OLT period (OR 2.10; 95% CI: 1.19–3.73; *p* = 0.011) [33]. Likewise, in another retrospective study, a MELD score > 9 was shown to be an independent predictor of acute kidney injury occurrence in the early post‐OLT period (OR 1.05; 95% CI: 1.01–1.09; *p* = 0.021) [34]. In a prospective study higher median MELD score was associated with higher risk of acute kidney injury in the post‐OLT [35]. It is worth mentioning that approximately half of our ARC OLT recipients had multiple episodes during the 30‐day ICU stay, whose occurrence was associated with lower illness severity in terms of SOFA score. This is in agreement with studies carried out in other settings of critically ill patients [4, 36, 37, 38].

Importantly, the concordance between the true ARC prevalence verified by means of the mCLCr and the presumed one estimated by means of the three different eGFR equations was quite poor. This finding is consistent with those of other studies conducted in different types of special ICU populations [9, 31, 39]. Regarding this, it should not be overlooked that the formulas to calculate eGFR are generally unreliable for predicting ARC, as they were designed to detect renal dysfunction rather than hyperfiltration. Consequently, this strongly supports the fact that even in the setting of critical OLT recipients, the mCLCr is an absolute need for properly detecting subjects with ARC.

It should not be overlooked that in approximately one‐fourth of the ARC episodes the mCLCr was even > 200 mL/min, namely a status potentially having a remarkable impact on fastening the renal elimination of hydrophilic drugs, including the BLs. In agreement with this and with the findings of studies carried out in other special populations of critically ill patients [13, 40], ARC was found to be the only factor associated with early aggressive PK/PD target non‐attainment among the subset of critical OLT recipients receiving standard dosages of BLs by CI for treating early posttransplant Gram‐negative infections (in as much as one‐third of ARC cases). This may support the contention that, in early posttransplant Gram‐negative infections, even delivering BLs by CI, namely the most valuable strategy for maximizing the likelihood of aggressive PK/PD target attainment under standard dosages, could not always suffice for protecting the OLT recipients experiencing ARC from therapeutic underexposure [41]. Importantly, we showed in a recent a 3‐year prospective, observational study that among 50 critical OLT recipients receiving CI BL therapy for early posttransplant Gram‐negative infections, aggressive PK/PD target non‐attainment was the only independent predictor of 30‐day resistance development (OR, 14.33 [95% CI: 1.46–140.53]; *p* = 0.02) [30]. This may call for the need of implementing as much as possible the TDM‐guided approach for personalizing the CI BL therapy even in this setting of critically ill patients. Alternatively, it could justify the need of administering higher dosing regimens of BLs by CI from the very beginning of treatment of early posttransplant Gram‐negative infections in the specific subpopulation of critical OLT recipients experiencing ARC [42].

Limitations of our study should be recognized. The small sample size might limit the generalizability of the findings. Being the study design retrospective, the number of days with mCLCr varied between patients, because requirement was at the discretion of the attending intensivists. This could have biased the overall true total number of ARC episodes, and consequently the true prevalence of ARC in this scenario. The fact that aggressive PK/PD target attainment of CI BLs was assessable in less than 50% of cases might have biased the true proportion of non‐attainment. In addition, we could not rule out that delivering BLs by intermittent infusion rather than by CI could have increased the proportion of aggressive PK/PD target non‐attainment among ARC patients. Total BLs concentrations were measured, and the free fractions were only estimated based on the plasma protein binding reported in the literature without considering any specific adjustment based on each patient's plasma protein levels.

## 5 Conclusions

The findings showed that ARC may be a quite prevalent condition among critical OLT recipients, especially in the first posttransplant week, which could hinder early aggressive PK/PD target attainment in patients receiving standard dosing regimens of beta‐lactams, regardless of delivering by CI. Larger prospective studies should be warranted for hopefully confirming the findings.

## Author Contributions

*Conceptualization*: Milo Gatti and Federico Pea. *Methodology*: Milo Gatti, Riccardo De Paola, and Federico Pea. *Formal analysis*: Milo Gatti, Riccardo De Paola, and Cristiana Laici. *Data curation*: Milo Gatti, Riccardo De Paola, Matteo Rinaldi, and Cristiana Laici. *Writing – original draft preparation*: Milo Gatti and Riccardo De Paola. *Writing – review and editing*: Antonio Siniscalchi, Pierluigi Viale, and Federico Pea. All authors have read and agreed to the published version of the manuscript.

## Funding

The work reported in this publication was funded by the Italian Ministry of Health, RC‐2024‐2790678.

## Ethics Statement

The study was conducted according to the guidelines of the Declaration of Helsinki and approved by the Ethics Committee (No. 308/2021/Oss/AOUBo on May 17, 2021, EM232–2022_308/ 2021/Oss/AOUBo on March 16, 2022, EM449–2023_308/ 2021/Oss/AOUBo on April 17, 2024, and CE AVEC: 272/ 2024/Sper/AOUBo on May 16, 2024). Informed written consent was waived due to the retrospective and observational nature of the study.

## Conflicts of Interest

Milo Gatti received personal fees from Angelini, AdvanzPharma, and Viatris. Pierluigi Viale has served as a consultant for Biomerieux, Gilead, Merck Sharp & Dohme, Nabriva, Nordic Pharma, Pfizer, Thermo‐Fisher and Venatorx, and received payment for serving on the speaker's bureau for Correvio, Gilead, MerckSharp & Dohme, Nordic Pharma, and Pfizer. Federico Pea participated in the speaker bureau for Angelini, BeiGene, Gilead, InfectoPharm, Menarini, Merck Sharp & Dohme, Pfizer, and Shionogi, and in advisory boards for BeiGene, Merck Sharp & Dohme, Pfizer, and Viatris. The other authors declare no conflicts of interest.

## Supporting information




**Supporting Information**: tid70237‐sup‐0001‐figureS1.tif

**Supporting Information**: tid70237‐sup‐0002‐figureS2.tif

**Supporting Information**: tid70237‐sup‐0003‐figureS3.tiff

**Supporting Information**: tid70237‐sup‐0004‐SuppMat.docx

## Data Availability

All data and materials generated during the current study are available from the corresponding author on reasonable request.
