# Supplementary material for: ARC in Critical Ill OLT Recipients: True Prevalence, Risk Factors, and Impact on Early Aggressive PK/PD Target Non‐Attainment of CI Beta‐Lactam Therapy for Posttransplant Gram‐Negative Infections
Source: Transpl Infect Dis. 2026 May 7;28(5):e70237. doi: 10.1111/tid.70237 (PMC13638763; doi:10.1111/tid.70237)
Supplement: Supplementary file 1 [file TID-28-e70237-s001.docx]

**Supplementary Materials**

**Supplementary Table 1 -** Methods for measuring and for estimating the Creatinine Clearance (mL/min)

**Supplementary Table 2 -** Univariate analysis comparing severity of clinical conditions in ARC vs. non-ARC episodes

**Supplementary Figure 1 –** Flowchart of patient inclusion and exclusion criteria. ARC: augmented renal clearance; ECPA: expert clinical pharmacological advice; ICU: intensive care unit; OLT: orthotopic liver transplant; TDM: therapeutic drug monitoring.

**Supplementary Figure 2 –** Timeline of the episodes of augmented renal clearance (ARC) experienced in the 30 post-transplant days period by 26 orthotopic liver transplant (OLT) recipients. Each row identifies a single OLT recipient. Green boxes identify the days with documented ARC (namely measured creatinine clearance (mCRC_L_) >120 mL/min/1.73 m² in females and >130 mL/min/1.73 m² in males), grey boxes those with mCLCr non-meeting ARC criteria, white boxes those with no mCLCr assessment.

**Supplementary Figure 3 –** Flowchart of OLT recipients receiving TDM-guided CI beta-lactam empirical and/or targeted treatment courses. ARC: augmented renal clearance; CI: continuous infusion; OLT: orthotopic liver transplant; TDM: therapeutic drug monitoring.

| **Supplementary Table 1 -** Methods for measuring and for estimating the Creatinine Clearance (mL/min) | |
| --- | --- |
| **Method** | **Formula** |
| mCLCr | $\frac{U_{CR} \times U_{Volume}}{S_{CR} \times T}$ |
| Cockcroft-Gault | $\frac{140-age}{S_{CR} \times WT/72}x\left[ 0.85 iffemale \right]$  *If BMI > 30 kg/m^2^, use adjusted weight (kg)*  *Adjusted weight (kg) = IBW + [(body weight – IBW) x 0.4]* |
| MDRD | $175\times S_{CR}^{-1.154}\times{Age}^{-0.203}\times\left[ 0.742 iffemale \right]$ |
| 2021 CKD-EPI | $\text{142 × min (S}$*_CR_/K) ^a^* $\times max$ *(S_CR_/K)^-1.200^* $\times{0.9938}^{age}\left[ \times1.012 iffemale \right]$ |
| BMI: body mass index; CKD-EPI: Chronic Kidney Disease Epidemiology Collaboration; IBW: ideal body weight in kg; K: adjustment factor for sex (0.7 for females and 0.9 for males); max: maximum of serum Cr/k or 1; min: minimum of serum Cr/k or 1; mCrCl, measured creatinine clearance; S_CR_ serum creatinine concentration; U_CR_: urinary creatinine excretion (mg/dL); U_volume_: urinary volume (mL); T: 24h collection time (1440 min); WT: weight; Age is expressed as years in all equations; in the Cockcroft-Gault equation, IBW (Kg) is calculated as 50 + 2.3 × (height in inches – 60) for males and as 45.5 + 2.3 × (height in inches – 60 ) for females; in the CKD-EPI equation, *a* is -0.241 for females and -0.302 for males. | |

**Supplementary Table 2 -** Univariate analysis comparing severity of clinical conditions in ARC vs. non-ARC episodes

| **Variables** | **ARC episodes**  **(n=54)** | | **Non-ARC episodes**  **(n=240)** | **Univariate analysis**  **p value** |
| --- | --- | --- | --- | --- |
| SOFA score [median (IQR)] | | 5 (4-6.75) | 6 (5-7) | **0.005** |
| Vasopressors [n (%)] | | 1 (1.7) | 13 (5.4) | 0.22 |
| Mechanical ventilation [n (%)] | | 9 (16.6) | 73 (30.4) | 0.15 |
| Serum creatinine levels (mg/dL) [median (IQR)] | | 0.45 (0.36-0.55) | 0.77 (0.62-1.00) | **< 0.001** |
| ARC: augmented renal clearance; IQR: interquartile range; SOFA: sequential organ failure assessment | | | | |
